# Harnessing Motivation to Alleviate Neglect

**DOI:** 10.3389/fnhum.2013.00230

**Published:** 2013-06-06

**Authors:** Charlotte Russell, Korina Li, Paresh A. Malhotra

**Affiliations:** ^1^Department of Psychology, Centre for Cognition and Neuroimaging, Brunel University, London, UK; ^2^Division of Brain Sciences, Imperial College London, London, UK

**Keywords:** neglect, motivation, reward, attention, extinction, striatum, music

## Abstract

The syndrome of spatial neglect results from the combination of a number of deficits in attention, with patients demonstrating both spatially lateralized and non-lateralized impairments. Previous reports have hinted that there may be a motivational component to neglect and that modulating this might alleviate some of the debilitating symptoms. Additionally, recent work on the effects of reward on attention in healthy participants has revealed improvements across a number of paradigms. As the primary deficit in neglect has been associated with attention, this evidence for reward’s effects is potentially important. However, until very recently there have been few empirical studies addressing this potential therapeutic avenue. Here we review the growing body of evidence that attentional impairments in neglect can be reduced by motivation, for example in the form of preferred music or anticipated monetary reward, and discuss the implications of this for treatments for these patients. Crucially these effects of positive motivation are not observed in all patients with neglect, suggesting that the consequences of motivation may relate to individual lesion anatomy. Given the key role of dopaminergic systems in motivational processes, we suggest that motivational stimulation might act as a surrogate for dopaminergic stimulation. In addition, we consider the relationship between clinical post stroke apathy and lack of response to motivation.

## Introduction

Spatial neglect is widely acknowledged to result from multiple component deficits, which are both spatially lateralized and non-lateralized (Leicester et al., 1969; Corbetta and Shulman, 2011). The majority of these impairments relate to dysfunction of attention (Mesulam, 1981) but it has been suggested that a further potentially influential component could relate to motivation (Mesulam, 1985; Ishiai et al., 1990). There has been a great deal of recent work examining how motivation, particularly in the form of reward, can affect attentional processes in healthy humans, across a diverse range of attention paradigms (Small et al., 2005; Della Libera and Chelazzi, 2006; Kiss et al., 2009; Hubner and Schlosser, 2010). This research has shown, for example, that motivationally salient stimuli are less susceptible to the attentional blink (Raymond and O’Brien, 2009) and that rewarding targets leads to greater priming in visual search (Kristjansson et al., 2010). Here we review studies of how motivational processes can modulate pathologically impaired attention in patients with spatial neglect. We examine the effects of monetary reward, music, task instruction, and emotionally “negative” motivating influences, as well as speculating how these may be incorporated into clinical strategies. Moreover, we examine how these factors may relate to the drug therapies that have been trialed for patients with neglect, and how their effects might inform our understanding of how individual treatments work.

## Reward

There has been a great deal of work examining the effects of reward on selective attention, a process that is profoundly affected in patients with neglect (Bagurdes et al., 2008; Hubner and Schlosser, 2010; Anderson et al., 2011). In one of the earliest of these studies, Della Libera and Chelazzi (2006) examined the effects of associating individual targets during a search task with monetary value, and found that the efficacy of selective attention could be modulated by rewarding feedback. Further studies by these and other authors have consistently demonstrated that associating visual stimuli with monetary incentive can lead to improved task performance. There have been two distinct possibilities proposed for the mechanism by which reward exerts its effect on attention. First, reward might act as a motivation for the top-down strategic control of attention or, alternatively, reward delivery can directly alter the processing of specific stimuli by increasing their attentional priority in a more bottom-up manner (Chelazzi et al., 2012). Furthermore, it is possible that both these mechanisms might simultaneously be activated in some circumstances.

Following on from this research, and anecdotal reports that providing monetary incentive improves neglect (Mesulam, 1985), we went on to systematically evaluate the effects of anticipated monetary reward in a group of 10 patients with spatial neglect secondary to right hemisphere stroke (Malhotra et al., 2013). We adapted a standard cancellation task replacing target stimuli with high value (£1 coins) or no-value (metal buttons) targets on Reward and No-Reward variants of the cancellation search task respectively. Patients were informed that they would receive £1.00 for each target canceled in the Reward task but they were simply instructed to cancel all button targets in the No-Reward task without mention of reward. Patients completed the two variations of cancellation task in a first session and then were given monetary incentive and informed that the amount was based on their performance on the Reward task. After this session, patients returned on a separate day and completed the two tasks again. In this second session performance was significantly improved in the Reward task *only*. This improvement was evident both when examining performance across the entire search array and also when contralesional cancellations were examined separately.

In this paradigm, only being informed that reward would be received was not sufficient for improvement as there was no difference in the two conditions in the first session. However, receipt of incentive and relevant feedback led to improved cancellation. This is in keeping with previous studies where participants either took part in a training session, giving them time to associate stimuli with reward, and/or received online feedback during task performance (Della Libera and Chelazzi, 2006; Engelmann et al., 2009; Kiss et al., 2009; Anderson et al., 2011).

Two patients showed no response to reward, and lesion subtraction showed that the principal brain area damaged in these two patients but intact in those patients that did respond, was the striatum. Although it is possible that attentional response to motivation may be disrupted by secondary or tertiary results of the brain injury itself, such as anosognosia or depression, respectively, this finding is consistent with the known importance of striatal structures in reward processing (O’Doherty et al., 2004), and also potentially sheds light on animal studies showing that experimentally induced neglect is more severe and less likely to recover when cortical damage is accompanied by striatal dysfunction (Van Vleet et al., 2003; Christakou et al., 2005). As we discuss in the next section, it may also increase our understanding of the variable responses to treatment that have been observed in pharmacological studies in neglect.

As neglect is not a unitary disorder, and results from the interaction of a number of deficits (Husain and Rorden, 2003; Hillis, 2006; Bartolomeo, 2007), there are a number of possible avenues for reward’s influence on neglect in our study. One explanatory mechanism could be through heightened arousal secondary to administration of financial reward, as increased arousal has been shown to enhance spatial awareness in neglect (Robertson et al., 1998) and the rewarding stimuli employed in this study have been associated with galvanic skin response changes (Pessiglione et al., 2007). This suggests that reward could have led to increased arousal and reduced neglect, but only during the Reward condition in the second session.

Another potential mechanism for the effects of reward could be through increasing target salience and modulated processing of high reward stimuli (Bays et al., 2010). Evidence suggests that association with reward affects stimulus salience, even when detrimental to task performance (Anderson et al., 2011; Della Libera et al., 2011). After Session 1, following incentive gain and performance feedback, the relative salience of all the £1 targets may have been greater, leading to patients finding targets that they were previously unable to mark. As a result it is possible that reward’s effects were mediated via arousal, modulation of target salience, or a combination of these mechanisms. Further work is required to evaluate whether reward acts via both, or only one of these routes, but, from the evidence above, it is possible that its incorporation into behavioral therapies for neglect may be of significant benefit (Robertson, 2013), perhaps particularly if it is associated with a functional task goal (Wu et al., 2001).

## Pleasant Music

An alternative means of inducing positive motivation is through the use of enjoyable music. It has been shown by Sarkamo et al. (2008) that listening to music has significant effects on cognition following stroke. These authors found that patients who had suffered middle cerebral artery stroke had better recovery of verbal memory and focused attention if they were regularly listening to their preferred music when compared to patients who were listening to audiobooks or a control group who were receiving standard rehabilitation alone. This result is in keeping with work in healthy subjects showing that listening to enjoyable music can improve cognitive performance in a number of domains (Rauscher et al., 1993; Thompson et al., 2005; Rowe et al., 2007). Preferred music’s effect on impaired visual attention has been more directly assessed in a study by Soto et al. (2009), where they examined the effects of pleasant music in three patients on an experimental visual extinction paradigm. They found that when patients listened to music that they preferred, they were better able to identify contralesional targets as compared to when they were listening to unpreferred music, or during a silent condition. In a separate experiment, they examined whether listening to preferred music was associated with increased arousal, which has been shown to improve awareness in neglect patients (Robertson et al., 1998), and found that that this was not the case, suggesting that the improvement was not via an arousal mechanism. This experimental work has been followed by more recent studies looking at the effects of music using standard clinical tasks (Chen et al., 2013; Tsai et al., 2013). In particular, Chen et al. (2013) examined the effects of pleasant music on neglect in a group of 19 patients and found it to improve visual search but to have no effect on line bisection. Moreover, they also observed a significant increase in leftward eye movements in comparison to control conditions. These authors speculated that listening to pleasant music might be more likely to affect performance on tasks requiring global visuospatial attention processing over the whole visual field rather than tasks such as line bisection involving a single object.

In their recent review of the effects of music listening on function after stroke, Sarkamo and Soto (2012) suggest that a possible mechanism for the effects of music on visual awareness is via activation of the mesolimbic dopaminergic reward system, which is in keeping with evidence that emotional arousal whilst listening to music is associated with endogenous dopamine release in striatal structures (Salimpoor et al., 2011). Such an explanation would also be consistent with the effects of monetary reward on neglect, the key role of dopamine in reward processing (Zald et al., 2004), and our own observation that reward did not lead to a reduction of neglect in patients with striatal damage (Malhotra et al., 2013). Together, these findings raise the intriguing possibility that positive motivation, in the form of music and anticipated monetary reward may act via endogenous dopamine release. Dopaminergic stimulation has previously been used as a possible treatment in neglect, but with varying results, and even where positive effects have been found these have not been observed in all treated individuals (Fleet et al., 1987; Geminiani et al., 1998; Grujic et al., 1998; Hurford et al., 1998; Barrett et al., 1999; Mukand et al., 2001; Gorgoraptis et al., 2012). It is possible that positive motivation, as described above, may act as a surrogate for dopaminergic therapy, and help predict good candidates for dopaminergic treatment. Further work is necessary to explore these issues further, and to systematically examine the anatomical substrates that are necessary for attentional responses to positive motivation and effective exogenous dopaminergic [and cholinergic (Rice and Cragg, 2004)] stimulation.

## Task Instruction and Sequence Completion

An intriguing slant on improving motivation has been provided by Ishiai et al. (1990), who reported a possible motivational component to impaired search performance in neglect after investigating the effect of numbering targets rather than solely canceling them during a search task. They found that numbering significantly improved search and reduced neglect on such a task, and suggested that the process of numbering increased motivation to find more targets and complete the task. When participants carried out a third cancellation session without numbering, neglect increased again, suggesting that their observation was not due to a practice effect. In another study, addressing neglect during object copying, Ishiai et al. (1997) showed that performance when copying a drawing improved significantly when participants were instructed to arrange items around a central circle rather than to directly copy an example, although both tasks required an identical response. These authors suggested that alteration of the instruction may have led to increased motivation during task performance. These methods of utilizing simple changes in task instruction to improve performance is potentially crucial when considering how harnessing motivation might improve neglect, and highlights the need for careful consideration of task instructions when implementing therapy for patients.

## Negative Emotional Motivation

The work described so far has attempted to employ positive motivation and the induction of positive mood in order to reduce neglect. However, there is a long research history of the converse – that is the effect of negatively valent emotional stimuli both on attention in healthy individuals, and in studies demonstrating preserved processing of these forms of stimuli in patients with neglect and extinction. This work is highly relevant here as this alternative form of motivation may recruit different mechanisms to those involved with positive motivational stimulation, thereby enabling therapies that are potentially suitable for alternative groups of patients, with different underlying pathological neuroanatomy.

Numerous studies have demonstrated that emotionally valent stimuli, such as faces or emotive words, require less attention to be processed, or under some circumstances, appear to be processed when outside the focus of attention and with consequent effects upon the eventual distribution of attention (Ohman, 1986; Pratto and John, 1991; Stormark et al., 1995; White, 1995; Bradley et al., 1997; Eastwood et al., 2001; Ro et al., 2001; Lavie et al., 2003). This preferential processing of emotive stimuli is particularly strong for negative stimuli such as fearful or unhappy faces (Whalen et al., 1998; Eastwood et al., 2001).

There is now a considerable body of work investigating the effects of emotion-evoking stimuli on attentional deficits in brain-injured patients with visuospatial neglect. A landmark study by Marshall and Halligan (1988) demonstrated the powerful effect of emotionally valent content, such that contralesional information – for which the patient remained entirely unaware – nevertheless influenced explicit decision making. More recently, Vuilleumier and Schwartz (2001b) have shown that contralesional detection on bilateral simultaneous stimulation trials is better for faces rather than shapes, and also better for expressive, whether happy or angry, rather than neutral faces in patients with extinction. In addition, the same authors have shown that fear-related stimuli are more likely to be detected compared to neutral stimuli (Vuilleumier and Schwartz, 2001a), when presented in the contralesional field of patients who exhibited left visual extinction, even when the stimuli are well-matched in low-level visual properties.

These effects have also been observed using versions of standard clinical tasks and during visual search. Tamietto et al. (2005) found that cueing patients with unilateral left cues was significantly better at reducing the rightward bias in line bisection when the cues, although task-irrelevant, were represented by emotional as opposed to neutral faces. Similarly, visual search for emotional left-sided targets amongst distractors has been shown to more efficient, with a greater number detected and with faster reaction times, than for their neutral counterparts (Lucas and Vuilleumier, 2008). Together, these observations suggest that the emotional valence of stimuli in neglected hemispace might be implicitly processed, to a great enough degree that these stimuli can subsequently bias the deployment of spatial attention and encourage motor behaviors into left-sided space. Intriguingly, such findings have not been restricted to the visual modality, and comparable results have been reported for patients with auditory extinction, who demonstrate a reduction in their lateral deficit in the presence of contralesional, emotionally significant, vocal stimuli relative to neutral utterances (Grandjean et al., 2008).

It has been proposed that intact visual pathways to the ventral temporal lobe and amygdala might mediate these distinct mechanisms of emotional attention (Morris et al., 2001; Vuilleumier, 2005). Grabowska et al. (2011) attempted to examine the neural correlates underlying these processes using a variety of emotional and neutral stimuli presented unilaterally in either ipsilesional or neglected contralesional hemifields. In accordance with previous findings, emotional pictures presented in left visual field were reported more frequently than neutral images, thus modulating neglect. This correlated with increased activity in right parahippocampal gyrus and right anterior cingulate cortex. Amygdala activity was not reported even for emotional stimuli detected in right hemispace, which is in contrast to numerous studies that have suggested a crucial role in emotional processes for this structure. Current evidence suggests that a number of brain regions are involved in the capture of attention by emotional stimuli, such as orbitofrontal and anterior cingulate cortex (Vuilleumier et al., 2002; Pessoa and Adolphs, 2010; Schwabe et al., 2011) and further research will help to clarify the exact roles of the amygdala as well as these structures in the mediation of emotional effects upon impaired attention.

This perceptual advantage for emotionally valent stimuli has, very recently, been explored in the context of a rehabilitation tool for patients with neglect. Dominguez-Borras et al. (2013) have reported that following aversive conditioning to a specific visual stimulus, bilateral simultaneous trials involving these stimuli reduced left visual extinction in a patient with right parietal damage, as compared to responses to the same stimuli before conditioning. That is, the patient’s contralesional performance improved after a negative emotional significance association was learnt for some stimuli. Although this is a single case study, it introduces a potentially exciting concept for the use of affective strategies in the rehabilitation of neglect.

## Conclusion

**Figure 1:**
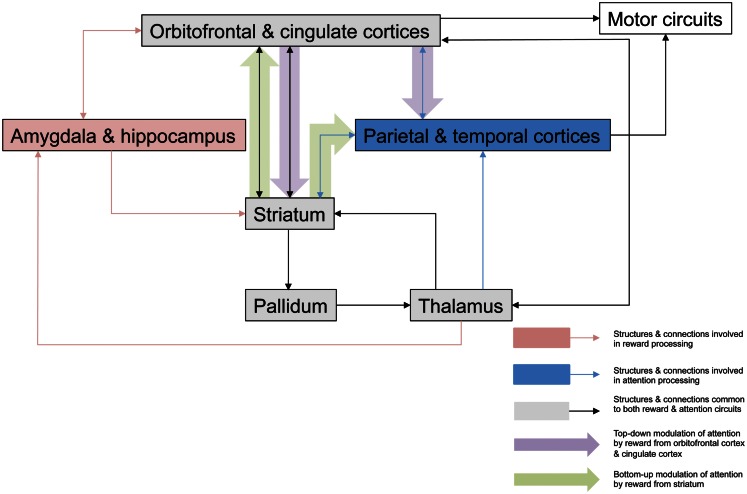
**Potential neuroanatomical pathways for motivation-attention interplay**. This outlines possible pathways involved in the interaction between motivation and attention that are critical for the actions of reward on spatial neglect. Mesulam (1999) proposed a large scale neurocognitive network with cortical foci in parietal cortex and cingulate cortex that is responsible for attention, and its organization allows very rapid surveys of perceptual representations, multiple coordinate systems, motor strategies, and motivational salience. Striatal neurons have been shown to be critical in reward processing (Haber and Knutson, 2010; Harsay et al., 2011), and striatal dopamine release in response to anticipated reward or amygdala activation secondary to emotionally relevant stimuli may have a direct modulatory effect on attentional signals being relayed to cortical areas for perceptual processing and representation, modulating the salience of such stimuli in a bottom-up manner. Reward may affect top-down control of attentional processes via dopaminergic projections from striatum to cingulate cortex to alter the salience of visual stimuli, with consequent behavioral effects, and there have been several studies demonstrating the role of the ACC in associating action with reward value (Bush et al., 2002; Williams et al., 2004; Small et al., 2005). Additionally this top-down modulation may also take place via orbitofrontal cortex, which appears to be involved in the enhanced processing of attentional information secondary to incentive (Mohanty et al., 2008). It is likely that these mechanisms are engaged to various degrees, according to the type of motivational stimulation, and the specific paradigm being employed.

In this review we have considered several mechanisms by which motivational influences might modulate awareness in patients with neglect, and described a number of studies that clearly demonstrate motivation’s considerable effects on attention (see Figure 1). Until recently, such studies were confined to experimental paradigms exploring this interaction, or anecdotal reports of individual patients improving following positive motivation. However, there have now been studies addressing these issues with more clinical tasks and employing motivational processes in the context of rehabilitation (Chen et al., 2013; Dominguez-Borras et al., 2013). Furthermore, there is very preliminary evidence to suggest that some motivational stimulation may act as a surrogate for pharmacological (in particular dopaminergic) therapy. However, there remains a great deal of work to be done in evaluating the precise mechanisms underlying these interactions and whether they rely on different neuroanatomical substrates. It has been shown that particular lesions appear to blunt motor responses to motivation, especially in the form of reward, and these are closely associated with clinical apathy (Schmidt et al., 2008; Adam et al., 2013), which is characterized by a lack of goal-directed behaviors due to loss of motivation (Marin, 1991). It is common following stroke (Starkstein et al., 1993), and has been found to be associated with disruption of basal ganglia circuits (Onoda et al., 2011). This is supported by the observation that apathy is a common feature of other pathological states associated with dysfunction of the frontal-basal ganglia system (Levy and Czernecki, 2006). Furthermore, in such cases, apathy is associated with a blunted neural response to motivation, especially in the form of reward (Czernecki et al., 2002; Lawrence et al., 2011). Although it has not yet been systematically investigated, this interplay between apathy, motivational response, and attentional impairments may be particularly important in determining outcome for many patients.

From a practical perspective, there remains considerable work to be done before specific therapeutic interventions can be recommended on the basis of well-controlled trials. However, it seems clear that patients with neglect are likely to perform better, if provided with motivational stimulation. This may involve access to music, in addition to the incorporation of a strong motivational component into goal-based therapy and the careful consideration of task instructions. In advance of the development of evidence-based guidelines, such measures could be adapted and applied by clinicians and therapists on an individual basis.

Finally, we have approached neglect as a unitary construct for the purposes of this review but it will be critical to examine experimentally the effects of motivation on the discrete component deficits of the syndrome, including spatial attention, sustained attention, and spatial working memory. After apparent recovery from neglect many patients are left with residual attentional impairments (Driver et al., 2004; Russell et al., 2010, 2012; Bonato, 2012), and it will be of considerable importance to examine whether these individuals also benefit from the exciting potential of motivational enhancement.

## Conflict of Interest Statement

The authors declare that the research was conducted in the absence of any commercial or financial relationships that could be construed as a potential conflict of interest.
